# Whole Genome Sequencing in Hypoplastic Left Heart Syndrome

**DOI:** 10.3390/jcdd9040117

**Published:** 2022-04-15

**Authors:** Jeanne L. Theis, Timothy M. Olson

**Affiliations:** 1 Cardiovascular Genetics Research Laboratory, Mayo Clinic, Rochester, MN 55905, USA; theis.jeanne@mayo.edu; 2 Department of Pediatric and Adolescent Medicine, Division of Pediatric Cardiology, Mayo Clinic, Rochester, MN 55905, USA; 3 Department of Cardiovascular Medicine, Mayo Clinic, Rochester, MN 55905, USA

**Keywords:** congenital heart disease, genetics, hypoplastic left heart syndrome, *MYH6*, whole genome sequencing

## Abstract

Hypoplastic left heart syndrome (HLHS) is a genetically complex disorder. Whole genome sequencing enables comprehensive scrutiny of single nucleotide variants and small insertions/deletions, within both coding and regulatory regions of the genome, revolutionizing susceptibility-gene discovery research. Because millions of rare variants comprise an individual genome, identification of alleles linked to HLHS necessitates filtering algorithms based on various parameters, such as inheritance, enrichment, omics data, known genotype–phenotype associations, and predictive or experimental modeling. In this brief review, we highlight family and cohort-based strategies used to analyze whole genome sequencing datasets and identify HLHS candidate genes. Key findings include compound and digenic heterozygosity among several prioritized genes and genetic associations between HLHS and bicuspid aortic valve or cardiomyopathy. Together with findings of independent genomic investigations, *MYH6* has emerged as a compelling disease gene for HLHS and other left-sided congenital heart diseases.

## 1. Introduction

**Figure 1:**
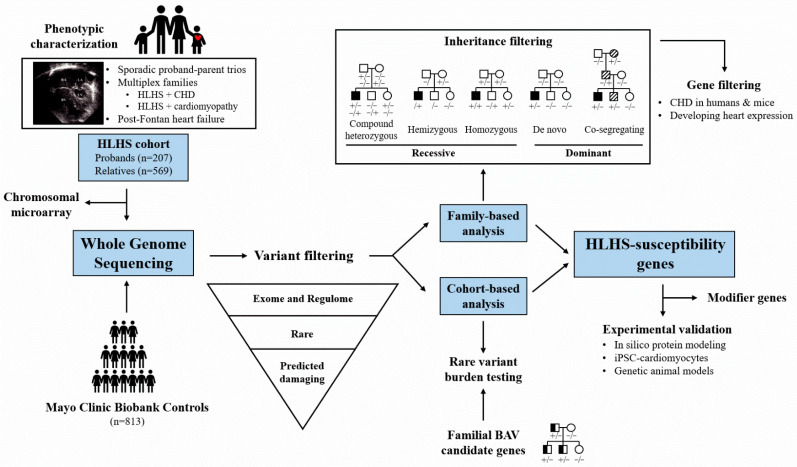
Whole genome sequencing and variant filtering algorithm. Depicted in the pedigrees are individuals with HLHS (black symbol); BAV (half symbol); cardiomyopathy or other CHD (diagonal stripes); family members without structural or myopathic heart disease (white symbol). Genotypes indicate presence (+) or absence (−) of a variant.

Hypoplastic left heart syndrome (HLHS) is a heritable but genetically complex disorder, for which a paucity of susceptibility genes has been identified and validated [1,2]. Its oligogenic underpinnings and predominantly sporadic presentation preclude genomic mapping and candidate gene strategies used in monogenic disorders. To discover HLHS candidate genes, we performed whole genome sequencing (WGS) in a cohort of non-syndromic HLHS probands, phenotypically characterized relatives [3], and a control group without personal or family histories of congenital heart disease (CHD) [4] (Figure 1). Chromosomal microarray was used to rule out large deletions and duplications not detected by WGS. The millions of rare coding and non-coding variants comprising individual exomes and regulomes, respectively, were filtered by variant and gene-based metrics, utilizing publicly available bioinformatics databases and tools (Table 1). Mendelian inheritance filters were applied in proband–parent trios (de novo and recessive alleles) and multiplex families (co-segregating dominant alleles), whereas rare variant burden testing of candidate gene bodies sought to identify enrichment in case versus control cohorts. Select candidate variants and genes were further evaluated by predictive or functional experimentation in model systems, including induced-pluripotent cell-derived cardiomyocytes (iPSC-CM) and genetically altered animals. Here, we provide synopses of our published HLHS-susceptibility gene investigations (Table 2). 

## 2. Compound Heterozygous NOTCH1 Mutations Underlie Impaired Cardiogenesis in a Patient with Hypoplastic Left Heart Syndrome 

Intrafamilial clustering of HLHS and other CHD, particularly left-sided malformations exemplified by bicuspid aortic valve (BAV), has been well established [5,6]. A research study design that includes screening echocardiography and WGS in relatives of HLHS probands can reveal asymptomatic CHD [3] and enable family-based inheritance filtering, dramatically reducing the number of potential candidate genes.

This study centered on a unique multiplex family comprising an HLHS proband, his mother with bicuspid pulmonary valve (a right-sided CHD), and a fourth-degree maternal relative with BAV [7]. We postulated that the proband’s severe developmental phenotype was due to inheritance of a variably expressed, CHD-segregating maternal variant and an incompletely penetrant paternal variant. Variant filtering revealed compound heterozygosity for rare, predicted-damaging missense variants in *NOTCH1*, a well-established gene for BAV [8]. Patient-specific induced pluripotent cells from the proband–parent trio provided functional evidence of perturbed NOTCH1 signaling and disorganized myofilaments. Mechanistically, these findings highlighted variant allele burden as a potential determinant of CHD. 

## 3. *CELSR1* Risk Alleles in Familial Bicuspid Aortic Valve and Hypoplastic Left Heart Syndrome

Like HLHS, BAV is a genetically complex and frequently sporadic disorder [9]. This study sought to identify an interfamilial genetic link between these left-sided CHDs [10]. WGS was first performed in six rare families, comprising three or more relatives with BAV. Filtering for rare, predicted-damaging, co-segregating variants in each family identified fifteen genes, previously associated with CHD in humans and mice. Postulating common genetic underpinnings between BAV and HLHS, the familial BAV candidate genes were interrogated in the HLHS and control cohort genomes. *CELSR1* was the only candidate found to have co-segregating variants in two BAV families and was also enriched for rare, predicted-damaging coding and regulatory variants in HLHS probands, predominantly inherited from a parent without CHD. Analyses of variants inherited from the other parent revealed compound, synergistic, or digenic heterozygosity in genes, comprising the planar cell polarity developmental pathway in 7 of 16 HLHS probands who inherited a *CELSR1* variant. These findings implicated *CELSR1* as a susceptibility gene for both BAV and HLHS, and *MYO15A* as an HLHS modifier; suggested a primary aortic valve developmental defect as a mechanism for HLHS; supported oligogenic, common-pathway underpinnings of sporadic HLHS, and highlighted the value of WGS for identification of non-coding variants that disrupt canonical transcription factor binding sites. 

## 4. Patient-Specific Genomics and Cross-Species Functional Analysis Implicate LRP2 in Hypoplastic Left Heart Syndrome 

This study described a multidisciplinary candidate gene discovery and prioritization platform in a family quintet, comprising an HLHS proband and his unaffected parents and siblings [11]. Familial WGS data were filtered for rare, predicted-damaging *de novo*, recessive, or loss-of-function variants and candidate genes were further prioritized based on differential mRNA expression in proband versus parental iPSC and iPSC-CM. The resulting ten candidate genes were then tested for aberrant cardiac phenotypes by heart-specific RNAi knockdown of orthologs in Drosophila, and proliferative defects by siRNA knockdown in healthy human iPSC-CM. Two genes, *LRP2* and *APOB*, each harboring compound heterozygous missense variants consistent with a loss-of-function disease mechanism, fulfilled all genetic, transcriptional profiling, and experimental model system requirements. Further, rare variant burden testing revealed enrichment for predicted-damaging missense variants in *LRP2* in HLHS versus control cohorts, and a hypoplastic ventricular phenotype was demonstrated in a zebrafish *lrp2* loss-of-function model. In summary, this study demonstrated a comprehensive, synergistic toolbox of strategies to analyze, prioritize, and validate candidate genes identified by WGS, in a single HLHS proband, culminating in deconvolution of a genetically complex disorder. Mechanistically, the findings implicated a myogenic, cellular proliferation defect as a basis for HLHS. 

## 5. Recessive *MYH6* Mutations in Hypoplastic Left Heart with Reduced Ejection Fraction 

Patients with HLHS who undergo successful staged surgical reconstruction to a Fontan circulation may develop latent right ventricular dysfunction. Postulating a genetic basis for poor outcome, this study focused on the families of five individuals with sporadic HLHS and right ventricular ejection fraction ≤40% after Fontan operation [12]. WGS was performed on the probands, parents, and siblings, and rare, predicted-damaging variants were filtered for *de novo* and recessive inheritance. Two of the five probands were found to be compound heterozygous for missense variants in *MYH6*, while parents and siblings who were heterozygous carriers had no structural or myopathic heart disease. Protein modeling predicted structural alterations by each amino acid substitution, affecting both head and tail domains. These findings reveal a recessive mechanism for sporadic HLHS and implicate perturbation of MYH6 in developmental arrest and latent myopathy of left and right ventricles, respectively. The association of rare, predicted-damaging variants in *MYH6* with HLHS and poor outcomes was replicated in an independent cohort [13].

## 6. Genetic Association between Hypoplastic Left Heart Syndrome and Cardiomyopathies 

Consistent with findings in other studies, echocardiographic screening revealed a spectrum of structural cardiovascular malformations in 27% of the HLHS probands in our cohort [3]. In addition, intrafamilial association of HLHS with dilated, hypertrophic, or restrictive cardiomyopathy was identified in three kindreds [14]. Postulating a shared genetic etiology of structural and myopathic heart disorders, WGS data were filtered for rare, predicted-damaging variants that co-segregated with HLHS and cardiomyopathy phenotypes in each family. Pathogenic or likely pathogenic variants in *MYBPC3* or *RYR2* were identified in all three families. Next, rare variant burden testing of 56 known cardiomyopathy genes demonstrated enrichment in *MYH6* in 23 HLHS probands, 12 of whom had either a *MYH6* variant co-segregating with familial CHD, compound heterozygosity, or synergistic heterozygosity with *FLNC*, which is also a cardiomyopathy gene. Collectively, these findings implicate genetic perturbation of sarcomeric proteins in HLHS pathogenesis, which may impose the risk of latent heart failure. Together with independent investigations demonstrating statistically significant genetic associations between *MYH6* and the Shone complex, coarctation of the aorta, and BAV [15,16], *MYH6* has emerged as a compelling disease gene for HLHS and other left-sided CHD. 

## 7. Future Directions

Our family-based HLHS cohort and WGS dataset provide a valuable resource for ongoing data mining and analysis to identify new candidate susceptibility and modifier genes, validate findings of other genetic studies of left-sided CHD, and further investigate the impact of gene variants on clinical outcomes. Since inception of our research investigations, over a decade ago, omics databases and bioinformatics tools have continued to evolve. New knowledge will lead to improved interpretation of non-coding variants and their impact on gene regulation. The emergence of robustly validated HLHS-susceptibility genes, such as *MYH6*, will inform prioritization of biological model systems to enable testing of gene–environment interactions, underlying arrested cardiac development and therapies to attenuate latent heart failure.

## Figures and Tables

**Table 1:** Bioinformatics databases and tools.

Resource	Application	Internet Website
Mouse Genome Informatics (MGI)	CHD phenotypes in gene knockout mice	www.informatics.jax.org
The Genome Aggregation Database (gnomAD)	Variant frequency, gene constraint	https://gnomad.broadinstitute.org
1000 Genomes (1000 G)	Variant frequency	https://www.internationalgenome.org/1000-genomes-browsers/index.html
Human Gene Mutation Database (HGMD)	CHD phenotypes linked to genetic variation in humans	http://www.hgmd.cf.ac.uk/ac/index.php
InterVar	Clinical interpretation of variants using guidelines established by the American College of Medical Genetics	https://wintervar.wglab.org/
Combined Annotation Dependent Depletion (CADD)	Integrated score used to predict deleteriousness of missense variants	https://cadd.gs.washington.edu/
RegulomeDB	Integrated score used to predict deleteriousness of variants in regulatory elements or non-coding regions	https://regulomedb.org/regulome-search/
MaxEntScan	Score used to predict deleterious nature of splice region variants	http://hollywood.mit.edu/burgelab/maxent/Xmaxentscan_scoreseq.html
Ensembl Variant Effect Predictor (VEP)	Consequence of variants on transcript or protein sequence	https://useast.ensembl.org/info/docs/tools/vep/index.html
Encode	mRNA expression in embryonic and fetal heart tissues	https://www.encodeproject.org/
Factorbook Position Weight Matrix (PWM)	Scored used to predict impact of variants on canonical transcription factor binding sites	https://www.factorbook.org/
University of Santa Cruz Genome Browser (UCSC)	Identification of transcription factor binding sites derived from CHIP-seq experiments	https://genome.ucsc.edu

**Table 2:** HLHS-susceptibility genes.

Gene Symbol	Protein	Ontology	Supportive Evidence	Other Reported Phenotypes
*MYH6*	Myosin heavy chain 6	conventional myosin of sarcomeric thick filament	1, 2, 3, 5, 7, 8, 9	A, B, C, D
*CELSR1*	Cadherin EGF LAG seven-pass G-type receptor	G-protein-coupled receptor	1, 2, 3	B, D
*NOTCH1*	Neurogenic locus notch homolog protein 1	transmembrane signaling protein	1, 2, 4, 7, 8	B, D
*LRP2*	LDL receptor related protein 2	multi-ligand endocytic receptor	2, 3, 4, 6	B, D
*MYBPC3*	Myosin binding protein C3	myosin binding protein of the sarcomeric thick filament	1, 2, 7, 8, 9	A, B, C
*RYR2*	Ryanodine receptor 2	calcium release channel of the sarcoplasmic reticulium	1, 7, 8, 9	A, C
*FLNC*	Filamin C	structural protein localized to Z-band and intercalated disks	*MYH6* modifier	A, C, D
*MYO15A*	Myosin XVA	unconventional myosin	*CELSR1* modifier	B, D

1 = co-segregation, 2 = compound heterozygosity, 3 = enrichment, 4 = iPSC-CM, 5 = protein modeling, 6 = genetic animal models, 7 = cardiovascular phenotype in humans (HGMD), 8 = cardiovascular phenotype in mice (MGI), 9 = pathogenic variant(s) (ACMG). A, cardiomyopathy; B, other CHD; C, arrhythmia; D, extra-cardiac disorder.

## Data Availability

Not applicable.
